# The VENUSS prognostic model to predict disease recurrence following surgery for non-metastatic papillary renal cell carcinoma: development and evaluation using the ASSURE prospective clinical trial cohort

**DOI:** 10.1186/s12916-019-1419-1

**Published:** 2019-10-03

**Authors:** Tobias Klatte, Kevin M. Gallagher, Luca Afferi, Alessandro Volpe, Nils Kroeger, Silvia Ribback, Alan McNeill, Antony C. P. Riddick, James N. Armitage, Tevita F. ‘Aho, Tim Eisen, Kate Fife, Axel Bex, Allan J. Pantuck, Grant D. Stewart

**Affiliations:** 1 Department of Urology, Addenbrooke’s Hospital, Cambridge, UK; 2 Department of Surgery, University of Cambridge, Cambridge, UK; 3 Department of Urology, The Royal Bournemouth and Christchurch Hospitals, Castle Lane East, Bournemouth, BH7 7DW UK; 4 Department of Urology, Western General Hospital, Edinburgh, UK; 5 Division of Urology, Department of Translational Medicine, University of Eastern Piedmont, Novara, Italy; 6 Department of Urology, Ernst-Moritz-Arndt-University, Greifswald, Germany; 7 Institute of Pathology, Ernst-Moritz-Arndt-University, Greifswald, Germany; 8 Department of Oncology, Addenbrooke’s Hospital, Cambridge, UK; 9 Astra Zeneca, Cambridge, UK; 10 Division of Surgical Oncology, Department of Urology, The Netherlands Cancer Institute, Amsterdam, The Netherlands; 11 Institute of Urologic Oncology, Department of Urology, University of California-Los Angeles, Los Angeles, CA USA

**Keywords:** Recurrence, Papillary, Prognosis, Adjuvant, Surveillance, Localised

## Abstract

**Background:**

The current World Health Organization classification recognises 12 major subtypes of renal cell carcinoma (RCC). Although these subtypes differ on molecular and clinical levels, they are generally managed as the same disease, simply because they occur in the same organ. Specifically, there is a paucity of tools to risk-stratify patients with papillary RCC (PRCC). The purpose of this study was to develop and evaluate a tool to risk-stratify patients with clinically non-metastatic PRCC following curative surgery.

**Methods:**

We studied clinicopathological variables and outcomes of 556 patients, who underwent full resection of sporadic, unilateral, non-metastatic (T1–4, N0–1, M0) PRCC at five institutions. Based on multivariable Fine-Gray competing risks regression models, we developed a prognostic scoring system to predict disease recurrence. This was further evaluated in the 150 PRCC patients recruited to the ASSURE trial. We compared the discrimination, calibration and decision-curve clinical net benefit against the Tumour, Node, Metastasis (TNM) stage group, University of California Integrated Staging System (UISS) and the 2018 Leibovich prognostic groups.

**Results:**

We developed the VENUSS score from significant variables on multivariable analysis, which were the presence of VEnous tumour thrombus, NUclear grade, Size, T and N Stage. We created three risk groups based on the VENUSS score, with a 5-year cumulative incidence of recurrence equalling 2.9% in low-risk, 15.4% in intermediate-risk and 54.5% in high-risk patients. 91.7% of low-risk patients had oligometastatic recurrent disease, compared to 16.7% of intermediate-risk and 40.0% of high-risk patients. Discrimination, calibration and clinical net benefit from VENUSS appeared to be superior to UISS, TNM and Leibovich prognostic groups.

**Conclusions:**

We developed and tested a prognostic model for patients with clinically non-metastatic PRCC, which is based on routine pathological variables. This model may be superior to standard models and could be used for tailoring postoperative surveillance and defining inclusion for prospective adjuvant clinical trials.

## Background

The 2016 World Health Organization classification recognises 12 major subtypes of renal cell carcinoma (RCC) with distinct morphologic, molecular and clinical features [1]. Papillary RCC (PRCC) is the second most common subtype and accounts for 15% of all cases [2]. PRCC is often subdivided into type 1 and 2, but studies did not confirm the independent prognostic value of PRCC type in localised disease [3, 4]. Compared with conventional clear cell RCC, PRCC is thought to have a more favourable prognosis in the non-metastatic stage [5], while patients with metastatic disease have worse outcomes than their counterparts [6]. However, although RCC subtypes differ on molecular and clinical levels, they are generally managed as the same disease, simply because they occur in the same organ and due to the fact that there is little data on the efficacy of available treatment options.

Prognostic factors are crucial for counselling, planning follow-up and selecting candidates for adjuvant trials. In general, the protocol of follow-up imaging studies reflects the risk, the site and the timing of recurrence, with more frequent imaging obtained in high-risk patients within the first years after surgery [7]. Further, it appears likely that patients at a higher risk for tumour recurrence are most in need of effective adjuvant therapies and should therefore be included in adjuvant trials [8]. In this regard, TNM stage has traditionally been used to establish the risk of tumour recurrence for all RCC subtypes, but has limited accuracy when used alone [9]. TNM has been supplemented by several additional independent prognostic factors such as grade and coagulative tumour necrosis [10, 11]; however, these prognostic models were often established for clear cell RCC only [12, 13] or all RCC subtypes [14, 15], disregarding the considerable proportion of patients with PRCC. Further, the prognostic models or modifications thereof were used to define inclusion criteria and to risk-stratify PRCC patients for adjuvant trials such as SORCE (NCT00492258) or ASSURE (NCT00326898, E2805) without previous validation, and others such as the 2018 Leibovich prognostic system [11] were single centre, not externally validated and not assessed for calibration or clinical net benefit. Thus, there is a great need for refinement of prognostic models in patients with PRCC, just as there is a need to establish a more specific approach to managing this second commonest subtype of RCC [16]. Here, we develop and evaluate a prognostic model for non-metastatic PRCC following curative surgery.

## Methods

### Development cohort

The development cohort included 556 bi-nephric patients, who underwent curative surgery for sporadic, non-metastatic (M0), unilateral PRCC at five international RCC centres between 2000 and 2016. Participating institutions obtained the required local institutional review board approval for retrospective analyses and provided required data-sharing agreements prior to initiation of the study.

Pre-operative clinical staging was performed through physical examination and computed tomography (CT) scans of the chest, abdomen and pelvis. None of the patients had preoperative systemic therapy, local radiotherapy or embolization, and all had complete macroscopic resection of disease. Two hundred eighty-three patients underwent radical nephrectomy, while 273 had a partial nephrectomy. A concomitant lymph node dissection was performed in 86 cases, with a median of five lymph nodes removed (range 1–32). Those who did not undergo a lymph node dissection (pNx) were clinically N0.

Pathological specimens were reviewed by specialised genitourinary pathologists at each institution. Tumours were staged clinically and pathologically according to the 2010 American Joint Committee on Cancer Tumour, Node, Metastasis (TNM) classification, and re-classified if older systems were used initially. Further recorded clinicopathological variables included nuclear grade [17], pathological tumour size, presence and extent of venous tumour thrombus, papillary type 1 or 2, sarcomatoid features and coagulative tumour necrosis. The presence of coagulative tumour necrosis was analysed microscopically and defined as homogeneous clusters and sheets of degenerating and dead cells. Clinicopathological variables were then used to assign prognostic groups according to UISS [18] and the 2018 Leibovich prognostic groups [11].

Postoperative follow-up was institution- and physician-dependent, but generally followed national and international guidelines at that time. In general, patients were seen at least every 3 to 6 months in year 1, every 6 months in year 2 and annually thereafter. Follow-up visits consisted of a physical examination, laboratory tests and CT scans of chest, abdomen and pelvis. Sixty PRCC recurrences were detected during a median follow-up of 50 months. Local recurrence, solitary metastasis and oligometastases defined as < 3 lesions at a single site were considered oligometastatic recurrent disease, which may be potentially curable by local therapies [19, 20]. There were 116 deaths, including 44 from PRCC.

### Independent cohort

ASSURE (Adjuvant Sunitinib or Sorafenib for Unfavourable REnal-cell carcinoma, ECOG-ACRIN E2805, NCT00326898) was a double-blind, placebo-controlled, randomised, phase 3 trial, which enrolled 1943 patients with completely resected non-metastatic RCC at high risk for recurrence. After a median follow-up of 5.8 years, there were no differences in disease-free survival for adjuvant sunitinib or sorafenib relative to placebo. The trial included patients with all RCC subtypes, including 150 with intermediate high-risk and very-high-risk PRCC according to modified UISS criteria [18]. There was central pathology review and standardised postoperative imaging, as outlined in the trial protocol [21]. We used the data of these patients for evaluation of our model. Of the 150 patients, 57 recurred and 33 died, including 31 with recurrent disease. Access to individual patient data was granted through Project Data Sphere (https://www.projectdatasphere.org/, ID 435).

### Statistical analysis

The primary outcome measure of this study was disease recurrence, which was calculated from the date of surgery to the date of first recurrence. The date of censoring for those alive was the date of last follow-up imaging. We defined recurrence according to the DATECAN renal cancer group, i.e. as local, regional recurrence or metastases or contralateral RCC, whichever occurred first [22]. Median follow-up was calculated using the inverse Kaplan-Meier approach.

We used Fine and Gray’s competing-risk regression to model the data, as this accounts for the competing risk of death without previous recurrence. Potential predictors of recurrence were analysed by univariable and multivariable models. Owing to the possibility of a nonlinear relationship between tumour size and recurrence, tumour size was also analysed using restricted cubic splines. We performed subsequent categorisation as a non-linear relationship was observed. After the univariable regression analysis, we fitted multivariable models. The initial multivariable model included all significant variables of the univariable analysis. Subsequently, the model was reduced through backward variable selection with the likelihood ratio criterion (inclusion/exclusion criteria: *p* ≤ 0.05/*p* > 0.1). A simple scoring algorithm was developed based on the final multivariable model, as described previously [23]. The coefficient for each variable was divided by the highest coefficient (i.e. lymph node metastasis), multiplied by 3 and rounded to the nearest integer.

We obtained various measures to analyse model performance. The overall performance was measured by the concordance index (*c* index), adapted for competing risks [24]. The internal validity of the score was evaluated by 500 bootstrap re-samples. We used calibration plots to compare predicted probabilities at 5 years to the observed frequencies (*pec* library in R by TA Gerts) [25]. A decision curve analysis (DCA) was applied to determine whether the clinical value of the newly derived model increased the net benefit over a realistic range of threshold probabilities [26]. *C* indices, calibration and DCA of the derived score and risk groups were compared with other risk group definitions, including the UISS, TNM and Leibovich groups.

In the ASSURE cohort, the score was calculated from the data obtained; however, tumour size and presence of venous tumour thrombus were not provided from Project Data Sphere. We obtained these variables indirectly in a subgroup of patients through trial inclusion criteria and logical vectors (i.e. T2 is greater than 4 cm, T1 and T2 have no tumour thrombus in renal vein or IVC). For the remaining missing variables, we used the Multivariate Imputation by Chained Equations algorithm to generate five imputations [27] using the *mice* library in R. *mice* creates multiple imputations (replacement values) for multivariable missing data using Gibbs sampling. The method is based on Fully Conditional Specification, where each incomplete variable is imputed by a separate model. We followed published guidelines for obtaining final estimates after multiple imputations [28], including Rubin’s rules to pool model parameters from the five imputed datasets [29].

We used STATA (Stata Inc., College Station, USA) for DCA and R (The R Foundation for Statistical Computing, Vienna, Austria; libraries *survival*, *rms*, *riskRegression*, *mice*, *pec*) for statistical analyses. Statistical testing was two-sided, and a *p* value < 0.05 was considered statistically significant.

## Results

### Characteristics

**Fig. 1:**
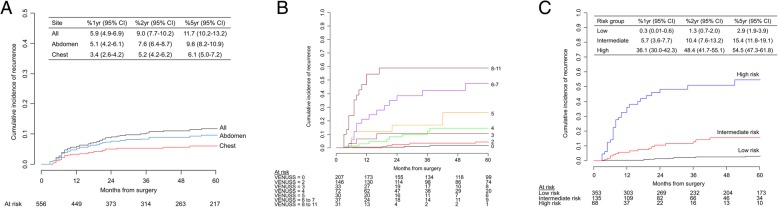
Cumulative incidence of disease recurrence of 556 who underwent surgery for non-metastatic papillary renal cell carcinoma and according to the first site of recurrence. Twenty patients first recurred simultaneously in the abdomen and chest and are included in both curves. **a** Overall, the cumulative incidence rate of disease recurrence was 11.7% at 5 years. The most frequent site of recurrence was the abdomen. **b** Cumulative incidence curve of disease recurrence according to VENUSS score. **c** Cumulative incidence curve of disease recurrence according to VENUSS group

Table 1 details characteristics of the 556 patients. Within a median postoperative follow-up interval of 53 months (SE = 3 months), disease recurrence was detected in 60 patients at a median of 12.5 months (IQR = 7–26.5). The cumulative incidence of recurrence was 5.9% (95% CI = 4.9–6.9%) at 1 year, 9.0% (95% CI = 7.7–10.2%) at 2 years and 11.7% (95% CI = 10.2–13.2%) at 5 years. The first recurrence was located in the chest in 30 (50.0%) and the abdomen in 49 patients (81.7%), with 20 patients (33.3%) developing their first recurrence in both the chest and abdomen. One patient recurred in a cervical node but had no concomitant metastases in chest and abdomen at that time. Figure 1 demonstrates the risk of recurrence for all patients and according to site. The first recurrence was oligometastatic in 26 (43.3%).

**Table 1:** Clinicopathological variables of 556 patients with unilateral PRCC treated with partial or radical nephrectomy (development cohort)

Variable	Category	
Age, years	Median	63
	IQR	54–70
Gender, *n* (%)	Female	145 (26.1)
	Male	411 (73.9)
T stage, *n* (%)	pT1	370 (66.5)
	pT2	61 (11.0)
	pT3	123 (22.1)
	pT4	2 (0.4)
N stage, *n* (%)	pNx/pN0	537 (96.6)
	pN1	19 (3.4)
Nuclear grade, *n* (%)	1 or 2	381 (68.5)
	3 or 4	175 (31.5)
Tumour size, *n* (%)	4.0 cm or less	285 (51.3)
	4.1 to 10.0 cm	219 (39.4)
	10.1 cm or greater	52 (9.4)
	Median	4.0
	IQR	2.7–6.3
Surgical margin, *n* (%)	Negative	525 (94.4)
	Positive	31 (5.6)
Venous tumour thrombus, *n* (%)	None	524 (94.2)
	Renal vein	15 (2.7)
	Cava	17 (3.1)
Perinephric/sinus fat invasion, no venous tumour thrombus, *n* (%)	–	93 (16.7)
Papillary type, *n* (%)	Type 1	227 (46.0)
	Type 2	266 (54.0)
Tumour necrosis, *n* (%)	–	254 (45.7)
Sarcomatoid features, *n* (%)	–	11 (2.0)
UISS, *n* (%)	Low/intermediate risk	335 (60.3)
	Intermediate high risk	141 (25.4)
	Very high risk	80 (14.4)
2018 Leibovich group, *n* (%)	Low (group 1)	322 (57.9)
	Intermediate (group 2)	102 (18.3)
	High (group 3)	132 (23.7)
TNM group, *n* (%)	I	369 (66.4)
	II	60 (10.8)
	III	125 (22.5)
	IV	2 (0.4)

### Prognostic factors and prognostic model

On univariable analysis, increasing tumour size (*p* < 0.001), T stage (*p* < 0.001), N stage (*p* < 0.001), nuclear grade (*p* < 0.001), presence of venous tumour thrombus (*p* < 0.001), tumour necrosis (*p* < 0.001), fat invasion (*p* < 0.001), papillary type (*p* < 0.001) and sarcomatoid features (*p* = 0.019) were all associated with an increased risk for recurrence (Table 2). With regard to tumour size, each 1-cm increase was associated with an 18% increase in the risk of recurrence (HR = 1.18, 95% CI = 1.12–1.23). The relationship between tumour size and recurrence was further analysed using restricted cubic splines. In this analysis, the risk for recurrence increased after 4 cm and subsequently reached a plateau at 10 cm (Additional file 3: Figure S3). Tumour size was therefore categorised at 4 and 10 cm. On the multivariable analysis, tumour size, T stage, N stage, grade and presence of venous tumour thrombus retained in the final model (Table 2).

**Table 2:** Univariable and multivariable Fine-Gray competing risks models predicting PRCC recurrence, while accounting for the competing risk of death without previous recurrence

		Univariable					Multivariable				
Variable	Category	Coeff	SE	SHR	95% CI	*p*	Coeff	SE	SHR	95% CI	*p*
Tumour size	0.1–4.0 cm	Reference					Reference				
	4.1–10.0 cm	1.64	0.34	5.15	2.60–10.2	< 0.001	1.00	0.38	2.73	1.31–5.68	0.007
	> 10 cm	2.52	0.40	12.38	5.63–27.2	< 0.001	1.09	0.49	2.97	1.14–7.74	0.026
T stage	pT1	Reference					Reference				
	pT2	1.47	0.41	4.36	1.94–9.8	< 0.001	0.69	0.44	1.99	0.84–4.69	0.12
	pT3	2.09	0.30	7.99	4.48–14.3	< 0.001	0.91	0.36	2.48	1.21–5.05	0.013
	pT4	4.22	0.28	68.3	39.4–118.4	< 0.001	1.15	0.63	3.15	0.92–10.8	0.068
N stage	pNx/pN0	Reference					Reference				
	pN1	2.55	0.36	12.8	6.37–25.9	< 0.001	1.49	0.46	4.42	1.78–11.0	0.001
Nuclear grade	G1 or G2	Reference					Reference				
	G3 or G4	1.55	0.26	4.73	2.85–7.87	< 0.001	0.93	0.30	2.53	1.42–4.53	0.002
Venous tumour thrombus	Absent	Reference					Reference				
	Renal vein	2.15	0.48	8.56	3.32–22.1	< 0.001	1.07	0.49	2.93	1.12–7.67	0.029
	IVC	2.44	0.35	11.5	5.77–23.0	< 0.001	0.95	0.45	2.57	1.08–6.16	0.034
Papillary type	Type 1	Reference									
	Type 2	1.07	0.31	2.91	1.58–5.34	< 0.001					
Fat invasion	Present	0.93	0.28	2.53	1.47–4.33	< 0.001					
Tumour necrosis	Present	1.01	0.27	2.74	1.61–4.67	< 0.001					
Sarcomatoid features	Present	1.33	0.57	3.77	1.24–11.5	0.019					
Surgical margin	Positive	0.45	0.46	1.56	0.63–3.86	0.34					

Data on papillary type 1 or 2 were available in 493 patients. On multivariable analysis, papillary type, tumour necrosis, fat invasion and sarcomatoid features were removed from the model during the backward selection process

The coefficients of this model were used to develop the continuous VENUSS (VEnous extension, NUclear grade, Size, Stage) score (Table 3, Fig. 1b), which ranges from 0 (lowest possible score) to 11 (highest possible score). Based on the VENUSS score, we defined three groups with regard to the risk of recurrence: low risk (0–2 points), intermediate risk (3 to 5 points) and high risk (6 points or greater) (Table 3, Fig. 1c). The low-risk group comprised 63.5% (*n* = 353), the intermediate-risk group 24.3% (*n* = 135) and the high-risk group 12.2% (*n* = 68) of patients. The 5-year cumulative incidence of recurrence ranged from 2.9% (95% CI = 1.9-3.9%) in the low-risk group to 54.5% (95%CI = 47.3–61.8%) in the high-risk group (Fig. 1c). Recurrences were oligometastatic in 91.7% (11/12) of recurring low-risk patients, 16.7% (3/18) of recurring intermediate-risk patients and 40.0% (12/30) of recurring high-risk patients (*p* < 0.001). In the 11 recurring low-risk patients, disease recurred in the abdomen in 9 (8 oligometastatic) and in the chest in 3 (all oligometastatic). In contrast, a preponderance of recurrent site was not observed in the intermediate- and high-risk groups.

**Table 3:** The VENUSS (VEnous extension, NUclear grade, Size, Stage) score and risk groups

Variable	Category	Score
Tumour size	4.0 cm or less	0
	4.1 cm or greater	2
T stage	pT1	0
	pT2	1
	pT3 or T4	2
N stage	pNx/pN0	0
	pN1	3
Nuclear grade	G1 or G2	0
	G3 or G4	2
Venous tumour thrombus	Absent	0
	Present	2
Risk group	Low risk	0–2
	Intermediate risk	3–5
	High risk	6 or greater

Points are assigned from each category, and the sum is used to determine the final score. Regression coefficient derived scores were similar for size categories 4.1 to 10 cm and 10.1 cm or greater, T3 and T4 and venous tumour thrombus present in the renal vein only or IVC, and therefore amalgamated into single categories. The minimum score is 0, the maximum score is 11

**Fig. 2:**
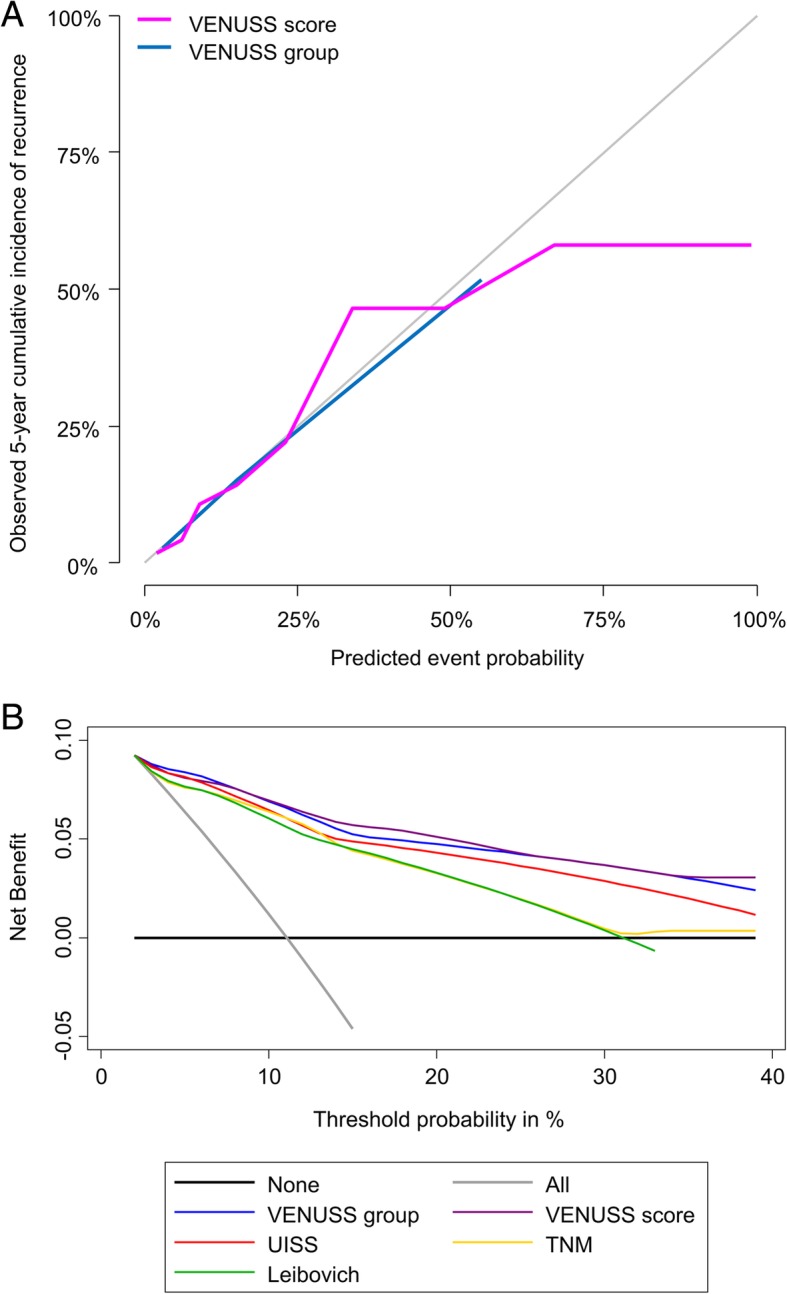
**a** Calibration of the VENUSS score and VENUSS group in predicting recurrence. The grey line represents the performance of an ideal prognostic model, while the purple and blue solid lines represent the performance of the VENUSS score and group, respectively. The graphs indicate good calibration. **b** Smoothed decision curves of VENUSS and other risk definitions predicting PRCC recurrence. Assuming that patients with PRCC would be treated differently (i.e. would be included in adjuvant trials), the net benefit of VENUSS is plotted against threshold probabilities and compared to UISS, TNM and Leibovich groups as well as the strategy of putting all or none into an adjuvant trial. Compared with UISS, TNM and the 2018 Leibovich prognostic group, VENUSS showed an improved net benefit between 10 and 40% threshold probability

Using bootstrapping for internal validation, the *c* index at 1 year, 2 years and 5 years was 91.4%, 87.2%, and 83.9% for the continuous VENUSS score, and 89.8%, 84.2% and 81.1% for the VENUSS group, respectively (Table 4). The predicted probability was comparable to the observed frequency of recurrence, indicating good calibration (Fig. 2a, Additional file 4: Figure S4). The corresponding competing-risks models are reported in Additional file 2: Table S2. Both the VENUSS score and the VENUSS group showed greater *c* indices at every time point than UISS, TNM and Leibovich groups (Table 4). The DCA demonstrated a moderate net benefit of the VENUSS score and group compared with the standard models in threshold probabilities between 10 and 40% (Fig. 2b).

**Table 4:** Comparison of *c* indices and bootstrap confidence intervals of VENUSS score, VENUSS group, UISS, TNM and Leibovich group in predicting recurrence at 1, 2 and 5 years

Cohort	Model	1 year	2 years	5 years
Development	VENUSS score	91.4 (85.5–95.0)	87.2 (83.8–90.7)	83.9 (78.7–88.6)
	VENUSS group	89.8 (84.4–93.5)	84.2 (79.5–88.7)	81.1 (75.5–86.5)
	UISS	88.6 (83.7–92.7)	82.9 (78.2–87.7)	79.3 (73.8–84.5)
	TNM	83.7 (78.4–88.4)	78.2 (72.8–82.6)	75.9 (70.8–80.4)
	2018 Leibovich group	78.7 (72.7–85.4)	78.2 (72.0–82.3)	76.5 (70.9–81.0)
ASSURE	VENUSS score	73.2 (64.5–80.4)	68.3 (61.3–76.1)	66.5 (60.5–73.7)
	VENUSS group	66.9 (58.7–74.0)	64.9 (59.6–70.8)	64.6 (57.8–69.8)
	UISS	64.2 (55.3–71.5)	63.7 (58.0–69.2)	62.5 (56.8–67.3)
	TNM	62.0 (54.0–69.3)	59.7 (53.8–66.6)	60.2 (53.9–65.8)
	2018 Leibovich group	61.3 (51.6–67.9)	60.0 (52.3–63.8)	58.2 (50.2–62.7)

### Performance in an independent cohort

Characteristics of the 150 PRCC patients enrolled in ASSURE are presented in Additional file 1: Table S1. Within a median follow-up of 61 months (SE = 2 months), 57 patients experienced disease recurrence. The estimated cumulative incidence of recurrence was 19.0% at 1 year, 30.9% at 2 years and 38.1% at 5 year, without a statistically significant difference across the three trial arms (*p* = 0.83).

The median VENUSS score was 5 (IQR 4–8, range 2–11), and 4% were grouped as VENUSS low risk, 50% as intermediate risk and 46% as high risk. Coefficients, SHR and 95% CI of competing risk models are shown in Additional file 2: Table S2. Both the VENUSS score and group showed better discrimination than UISS, TNM and Leibovich groups at every investigated time point (Table 4). All models were moderately well calibrated (Additional file 5: Figure S5A). The DCA showed a net benefit of these models in threshold probabilities of more than 20% (Additional file 5: Figure S5B). Additional file 6: Figure S6 shows cumulative incidence curves according to UISS, Leibovich group and VENUSS group.

## Discussion

In this study, we developed the VENUSS prognostic score for clinically non-metastatic PRCC, which is based on tumour size, T stage, N stage, presence of venous tumour thrombus and nuclear grade. The performance was further evaluated in an independent cohort of 150 high-risk PRCC patients from the prospective adjuvant ASSURE clinical trial. We show that the VENUSS score and the corresponding VENUSS groups may be superior to UISS, TNM and the 2018 Leibovich prognostic groups [11]. VENUSS may be used for patient counselling, follow-up planning and for prognostic stratification in adjuvant trials.

There has been no general consensus on how to best risk-stratify patients with PRCC following curative surgery. Guidelines advocate the use stratification systems such as UISS [30], which was developed on patients with all RCC subtypes; however, the majority of tumours were clear cell [14]. Although both PRCC and clear cell RCC share prognostic factors such as T stage and N stage, the individual contribution of each factor to the overall recurrence risk is different, and some factors such as tumour necrosis may not be prognostic in PRCC [11]. Some researchers used the TNM group [19], which does not appreciate additional prognostic factors such as venous tumour thrombus and only considers tumour size indirectly through T stage. Interestingly, prospective adjuvant trials such as ASSURE and SORCE used a modified UISS [21] or the 2003 Leibovich score [12] for defining inclusion and assessing baseline risk, both of which however were not validated in these patients.

Several PRCC prognostic models were published over the past years. A nomogram predicting disease-specific survival was developed and validated in 2010, but included both patients with and without distant metastases [10] and may therefore be of limited clinical utility. Buti et al. [31] developed the GRade, Age, Nodes and Tumour (GRANT) score from the ASSURE trial cohort for both clear cell and non-clear cell RCC. Recently, Leibovich et al. [11] published a prognostic model for PRCC, which is based on 607 surgically treated patients from the Mayo Clinic. Based on nuclear grade, fat invasion and the presence of venous tumour thrombus, the authors proposed three groups for recurrence and death from PRCC. The *c* index of this model was 77%, but calibration (i.e. comparing the predicted probability and the observed frequency) or clinical net benefits were not assessed [11]. In the present study, we compared VENUSS with other prognostic models, including UISS, TNM and the 2018 Leibovich prognostic groups. While the *c* index of the Leibovich prognostic groups was comparable with the original publication [11], VENUSS showed better discrimination in both the development and the ASSURE cohort. Of note, UISS was found to be superior to TNM and Leibovich prognostic groups. However, it is possible that both the UISS and the Leibovich prognostic groups showed a poorer performance than VENUSS as they were developed for different endpoints. Indeed, prognostic models are often used for different endpoints in clinical practice. For example, the ASSURE trial used the UISS (outcome of interest: overall survival), but the primary endpoint of ASSURE was disease-free survival.

Critically, our study included an independent cohort, which were the PRCC patients of the prospective adjuvant ASSURE clinical trial. The dataset was available from Project Data Sphere, which provides researchers the opportunity to conduct secondary analyses of prospectively collected trial data. In this analysis, discrimination and calibration were worse than in the development cohort, which is due to cohort composition. Indeed, two thirds of patients in the development cohort had stage I disease, compared to 10% of patients in ASSURE. While the development cohort included consecutive patients, ASSURE recruited from pre-screened patients with a higher risk of recurrence. Thus, although both cohorts included the same subtype of RCC, they were different in terms of the risk of recurrence due to the different distribution of prognostic factors. Subsequently, differences between study cohorts led to substantial differences in *c* indices and calibration, which in turn depend critically on variation of predictors [32]. As ASSURE included only patients at high risk of recurrence, there was little variation in predictors and thus lower discrimination and worse calibration, specifically in those with a lower risk of recurrence according to VENUSS. Thus, quality measures in the development and independent cohort cannot be compared directly, but VENUSS appeared to be superior to the other prognostic models.

An interesting observation was that the proportion of patients with oligometastatic recurrence was greater in high-risk than intermediate-risk patients. This finding has to be treated with reservation as the number of patients becomes low in each subgroup. While further validation is required, our data emphasise that patients with high-risk disease may benefit from close follow-up, as a considerable proportion of patients with oligorecurrent disease may be amenable to potentially curative salvage procedures.

An important benefit of VENUSS is that it is based on routine pathology and does not include clinical variables such as performances status or symptoms, which may be more subjective. There is little extra work for the reporting pathologist to assign the score and group. This can then be used for patient counselling and planning of follow-up.

We analysed one of the largest cohorts of non-metastatic PRCC, followed established research guidelines for prognostic modelling [33] and used an independent cohort to test the performance of VENUSS and to compare it do other risk group definitions. However, this study has a number of limitations, arising mainly from the retrospective character of the development cohort, missing candidate prognostic variables, as well as the possibility of not having picked up all recurrences. Firstly, the follow-up regimen was not standardised across centres, but generally followed international guidelines of the time. As the median follow-up was 53 months, it was not possible to present evidence beyond the 5-year landmark. Secondly, as the development cohort was retrospective, clinical and pathological data were reviewed locally rather than centrally. We feel that our results were not deeply hampered by this approach, as only standard clinical and pathological variables were analysed; however, we cannot exclude underreporting of pathological features. Our study represents a real-world scenario in which a central review is rarely performed, making the conclusions more generally applicable. Additionally, VENUSS and other definitions were also evaluated in an independent cohort from prospectively documented trial data, which may be considered the gold standard. Thirdly, it was not possible to adjust for multiple non-measured confounders, such as patient preference for follow-up imaging, imaging modalities, co-morbidity, symptoms, laboratory values and performance status, which were not available. However, the aim of this study was to provide a simple score based on routine pathological parameters. Papillary type 1 and 2 was only available in a subgroup of patients. It has been suggested that nuclear grade may be used as a surrogate for type [11], but there is no high-level evidence at present to support this approach. Additionally, some centres do no routinely grade PRCC. A proportion of type 2 PRCC may be hereditary leiomyomatosis and renal cell cancer (HLRCC), which may be another confounder given the highly aggressive nature of this disease. For this study, we only collected patients with documented sporadic PRCC, but cannot exclude that some patients may have had undocumented or undiagnosed HLRCC. As other groups [3, 4], the current study did not identify papillary type as a significant prognostic factor on multivariable analysis, but this may be due to the lack of central pathology review. This is also true for the presence of tumour necrosis and sarcomatoid features. It may be the case that the presence of both pathological features is not prognostic, but that a certain percentage is required to show statistical significance. Finally, we did not obtain data on treatment of recurrent disease, which was beyond the scope of this study. Instead, we focused on the time interval from surgery to detection of recurrence. Our proportion of patients with oligometastatic recurrent disease was comparable to other studies [19, 20], which supports the validity of our dataset. The current study reinforces the concept that, with routine follow-up imaging, oligometastatic and thus potentially curable disease is detected in a significant proportion of patients across all risk groups. Despite these limitations, our model may form the basis for follow-up risk stratification and inclusion criteria for adjuvant trials.

## Conclusions

We developed and tested a prognostic model for patients with clinically non-metastatic PRCC, which is based on routine pathological variables. This model may be superior to current standard models. This tool could be used for tailoring postoperative surveillance and defining inclusion into prospective adjuvant clinical trials.

## Supplementary information

**Additional file 1: Table S1.** Clinicopathological variables of the PRCC patients enrolled in ASSURE.

**Additional file 2: Table S2.** Competing risks models for VENUSS score, VENUSS, group TNM group and Leibovich group.

**Additional file 3: Figure S3.** Estimated restricted cubic spline function for tumour size versus the log hazard for tumour recurrence.

**Additional file 4: Figure S4.** Non-smoothed decision curves of VENUSS and other risk definitions predicting PRCC recurrence.

**Additional file 5: Figure S5.** Calibration and decision curve analysis of the VENUSS score, VENUSS group, UISS, TNM and Leibovich group in the ASSURE dataset.

**Additional file 6: Figure S6.** cumulative incidence of disease recurrence of patients with PRCC recruited into ASSURE according to UISS, Leibovich Score 2018 and VENUSS group.

## Data Availability

The datasets used and/or analysed during the current study are available from the corresponding author on reasonable request.

## Abbreviations

ASSURE: Adjuvant sorafenib or sunitinib for unfavourable renal carcinoma
C index: Concordance index
CT: Computed tomography
DCA: Decision curve analysis
IVC: Inferior vena cava
PRCC: Papillary renal cell carcinoma
RCC: Renal cell carcinoma
SHR: Subhazard ratio
TNM: Tumour, Node, Metastasis
UISS: University of California Integrated Staging System
