# Supplementary material for: The VENUSS prognostic model to predict disease recurrence following surgery for non-metastatic papillary renal cell carcinoma: development and evaluation using the ASSURE prospective clinical trial cohort
Source: BMC Med. 2019 Oct 3;17:182. doi: 10.1186/s12916-019-1419-1 (PMC6775651; doi:10.1186/s12916-019-1419-1)
Supplement: Supplementary file 1 — Additional file 1: Table S1. Clinicopathological variables of the PRCC patients enrolled in ASSURE. [file 12916_2019_1419_MOESM1_ESM.pdf]

Supplementary Table 1

Clinicopathological variables of the 150 PRCC patients enrolled in ASSURE (independent cohort).

Variable	Category	
Age – years	Median	57
	IQR	50-66
Gender - n (%)	Female	37 (24.7)
	Male	113 (75.3)
Type of nephrectomy	Radical	130 (86.7)
	Partial	20 (13.3)
T classification - n (%)	pT1	18 (12.0)
	pT2	53 (35.3)
	pT3	77 (51.3)
	pT4	2 (1.3)
N classification - n (%)	pNx/pN0	113 (75.3)
	pN1	37 (24.7)
Nuclear grade - n (%)	1 or 2	54 (36.0)
	3 or 4	92 (61.3)
	Unknown	4 (2.7)
UISS - n (%)	Intermediate high risk	83 (55.3)
	Very high risk	67 (44.7)
TNM group – n (%)	I	15 (10.0)
	II	45 (30.0)
	III	88 (58.7)
	IV	2 (1.3)
2018 Leibovich group – n (%)	Low (group 1)	29 (19.9)
	Intermediate (group 2)	33 (22.6)
	High (group 3)	84 (57.5)
Adjuvant therapy – n (%)	Placebo	59 (39.3)
	Sunitinib	39 (26.0)
	Sorafenib	52 (34.7)
