# Supplementary material for: The VENUSS prognostic model to predict disease recurrence following surgery for non-metastatic papillary renal cell carcinoma: development and evaluation using the ASSURE prospective clinical trial cohort
Source: BMC Med. 2019 Oct 3;17:182. doi: 10.1186/s12916-019-1419-1 (PMC6775651; doi:10.1186/s12916-019-1419-1)
Supplement: Supplementary file 2 — Additional file 2: Table S2. Competing risks models for VENUSS score, VENUSS, group TNM group and Leibovich group. [file 12916_2019_1419_MOESM2_ESM.pdf]

Supplementary Table 2

Continuous VENUSS score and risk group definitions according to VENUSS group, UISS, TNM group and Leibovich group in the development cohort (A) and the independent ASSURE cohort (B). The coefficients (coeff), standard errors (SE), subhazard ratios (SHR) and confidence intervals (95% CI) are derived from separate competing risks models. In the independent cohort, Rubin's rules were followed to obtain the reported pooled model parameters.

A

	**Category**	**Coeff**	**SE**	**SHR**	**95% CI**	***p***
VENUSS score	Continuous	0.485	0.046	1.62	1.48-1.78	<0.001
VENUSS group	Low risk	Reference				
	Intermediate risk	1.58	0.361	4.85	2.38-9.84	<0.001
	High risk	3.15	0.335	23.3	12.1-45.0	<0.001
UISS	Low/intermediate	Reference				
	Intermediate high risk	1.40	0.365	4.04	1.97-8.27	<0.001
	Very high risk	2.85	0.330	17.37	9.10-33.2	<0.001
TNM group	I	Reference				
	II	1.36	0.429	3.90	1.68-9.04	0.002
	III	2.09	0.294	8.09	4.55-14.4	<0.001
	IV	4.22	0.281	68.2	39.3-118.1	<0.001
Leibovich 2018	Low (group 1)	Reference				
	Intermediate (group 2)	1.11	0.412	3.03	1.35-6.80	0.007
	High (group 3)	2.21	0.316	9.10	4.90-16.9	<0.001

B

	**Category**	**Coeff**	**SE**	**SHR**	**95% CI**	***p***
VENUSS score	Continuous	0.238	0.058	1.27	1.13-1.42	<0.001
VENUSS group	Low/intermediate risk	Reference				
	High risk	1.11	0.28	3.04	1.75-5.27	<0.001
UISS	Intermediate high risk	Reference				
	Very high risk	0.95	0.27	2.58	1.52-4.41	<0.001
TNM group	I	Reference				
	II	0.62	0.75	1.86	0.43-8.06	0.41
	III-IV	1.39	0.70	4.03	1.02-16.0	0.047
Leibovich 2018	Low (group 1)	Reference				
	Intermediate (group 2)	0.13	0.46	1.14	0.46-2.80	0.78
	High (group 3)	0.68	0.37	1.97	0.96-4.08	0.07
