# Supplementary material for: The VENUSS prognostic model to predict disease recurrence following surgery for non-metastatic papillary renal cell carcinoma: development and evaluation using the ASSURE prospective clinical trial cohort
Source: BMC Med. 2019 Oct 3;17:182. doi: 10.1186/s12916-019-1419-1 (PMC6775651; doi:10.1186/s12916-019-1419-1)

Supplementary Figure 1

Estimated restricted cubic spline function for tumour size versus the log hazard for tumour recurrence. The risk for recurrence increased after 4 cm and subsequently reached a plateau at 10 cm. We used both cut-offs for further analyses.

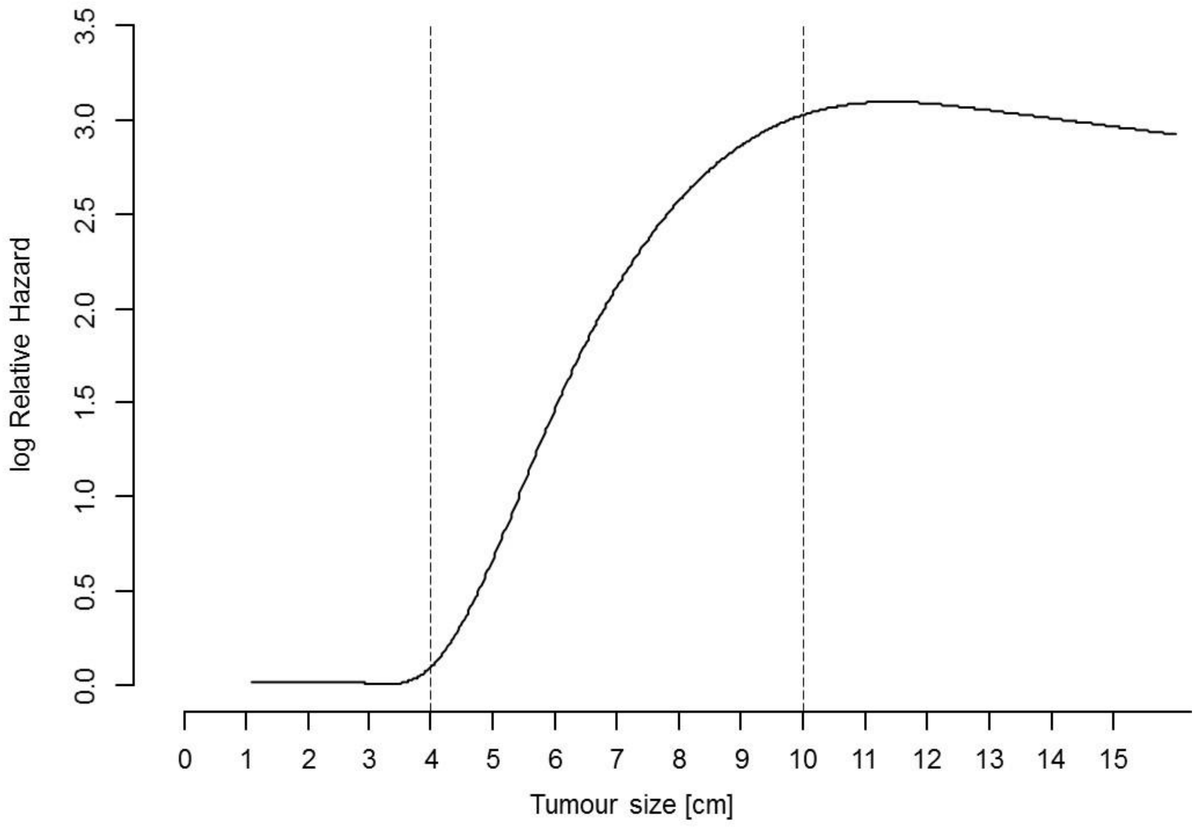

Supplement: Supplementary file 3 — Additional file 3: Figure S3. Estimated restricted cubic spline function for tumour size versus the log hazard for tumour recurrence. [file 12916_2019_1419_MOESM3_ESM.pdf]
