# Supplementary figures and images for: The VENUSS prognostic model to predict disease recurrence following surgery for non-metastatic papillary renal cell carcinoma: development and evaluation using the ASSURE prospective clinical trial cohort
Source: BMC Med. 2019 Oct 3;17:182. doi: 10.1186/s12916-019-1419-1 (PMC6775651; doi:10.1186/s12916-019-1419-1)

## Supplementary Figure 2

Non-smoothed decision curves of VENUSS and other risk definitions.

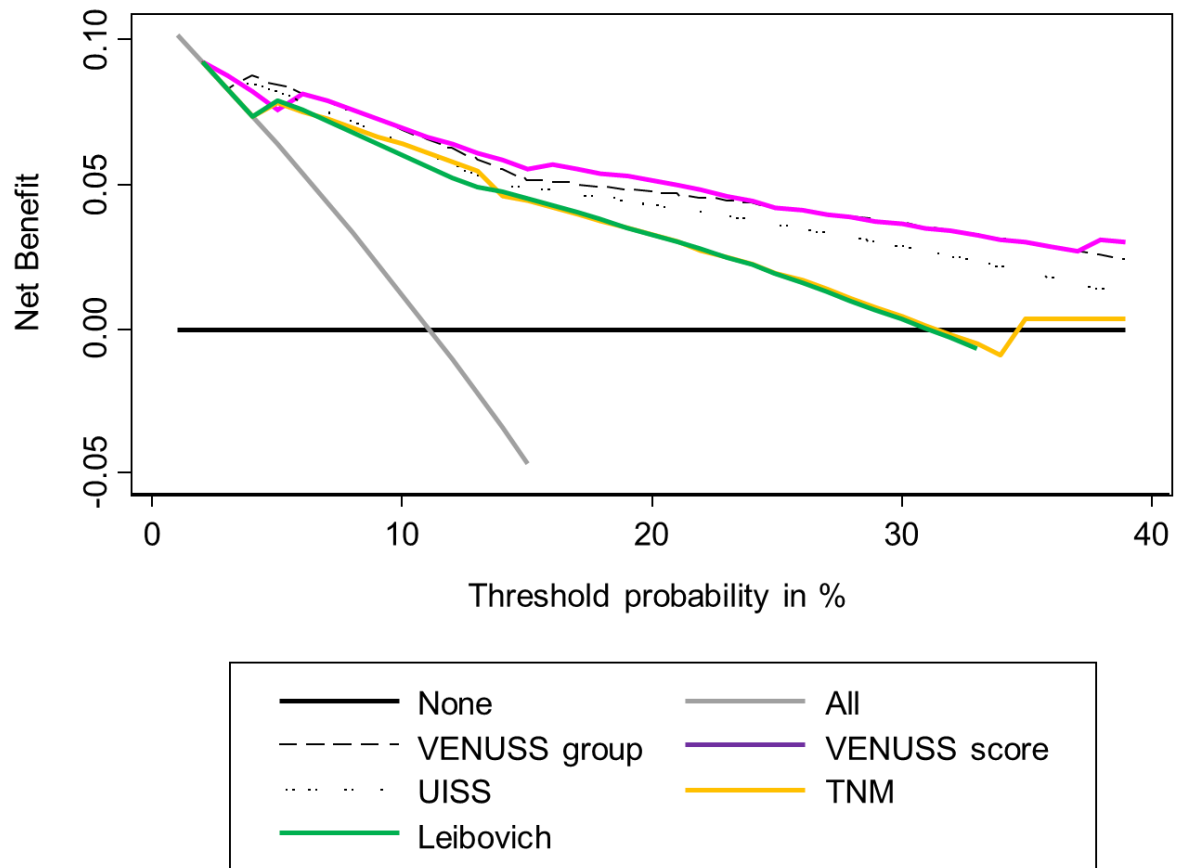

Supplement: Supplementary file 4 — Additional file 4: Figure S4. Non-smoothed decision curves of VENUSS and other risk definitions predicting PRCC recurrence. [file 12916_2019_1419_MOESM4_ESM.pdf]
