# Supplementary material for: The VENUSS prognostic model to predict disease recurrence following surgery for non-metastatic papillary renal cell carcinoma: development and evaluation using the ASSURE prospective clinical trial cohort
Source: BMC Med. 2019 Oct 3;17:182. doi: 10.1186/s12916-019-1419-1 (PMC6775651; doi:10.1186/s12916-019-1419-1)

Supplementary Figure 3

A - Calibration of the VENUSS score and VENUSS group in predicting recurrence in the ASSURE dataset. The grey line represents the performance of an ideal prognostic model, while the solid lines represent the performance of the VENUSS group, VENUSS score, UISS, TNM and Leibovich group respectively.

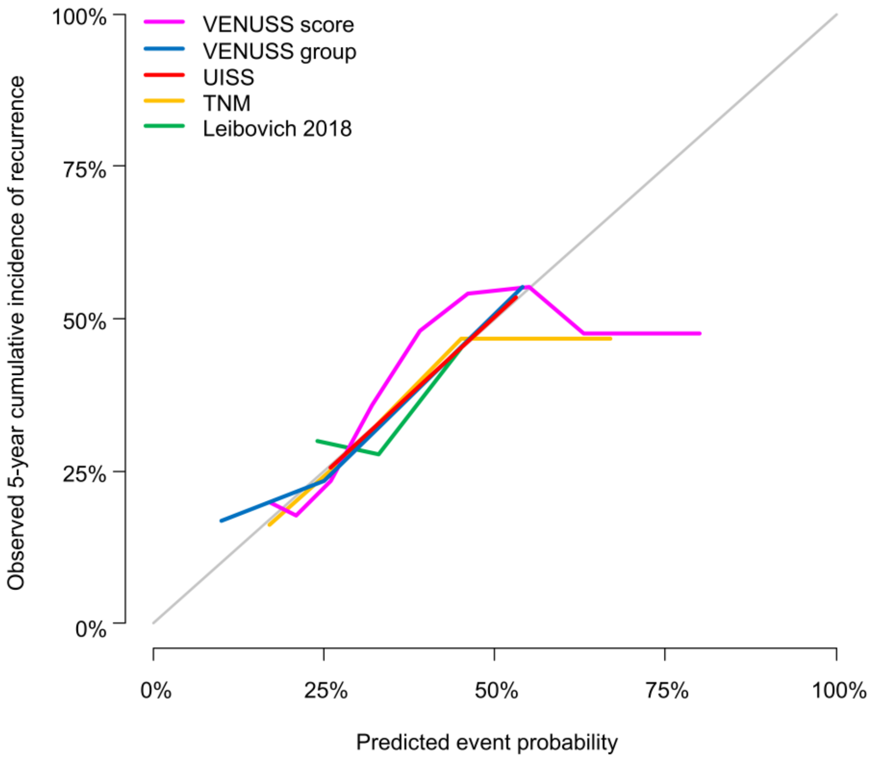

B

Smoothed decision curve analysis of the different prognostic models predicting PRCC recurrence. Both the VENUSS score and group showed a net benefit in threshold probabilities exceeding 20%.

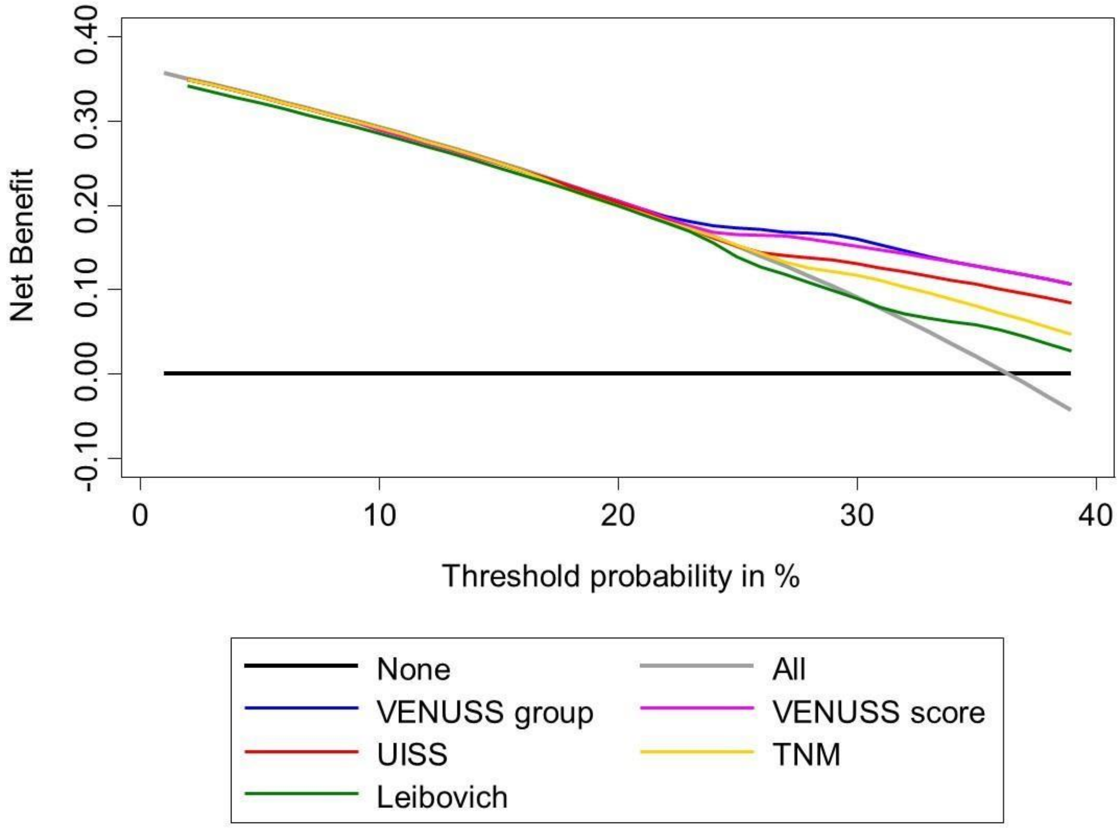

Supplement: Supplementary file 5 — Additional file 5: Figure S5. Calibration and decision curve analysis of the VENUSS score, VENUSS group, UISS, TNM and Leibovich group in the ASSURE dataset. [file 12916_2019_1419_MOESM5_ESM.pdf]
