# Supplementary material for: The VENUSS prognostic model to predict disease recurrence following surgery for non-metastatic papillary renal cell carcinoma: development and evaluation using the ASSURE prospective clinical trial cohort
Source: BMC Med. 2019 Oct 3;17:182. doi: 10.1186/s12916-019-1419-1 (PMC6775651; doi:10.1186/s12916-019-1419-1)
Supplement: Supplementary file 6 — Additional file 6: Figure S6. cumulative incidence of disease recurrence of patients with PRCC recruited into ASSURE according to UISS, Leibovich Score 2018 and VENUSS group. [file 12916_2019_1419_MOESM6_ESM.pdf]

Supplementary Figure 4

Cumulative incidence of disease recurrence of patients with PRCC recruited into ASSURE according to A – UISS, B – Leibovich group 2018, and C – VENUSS group.

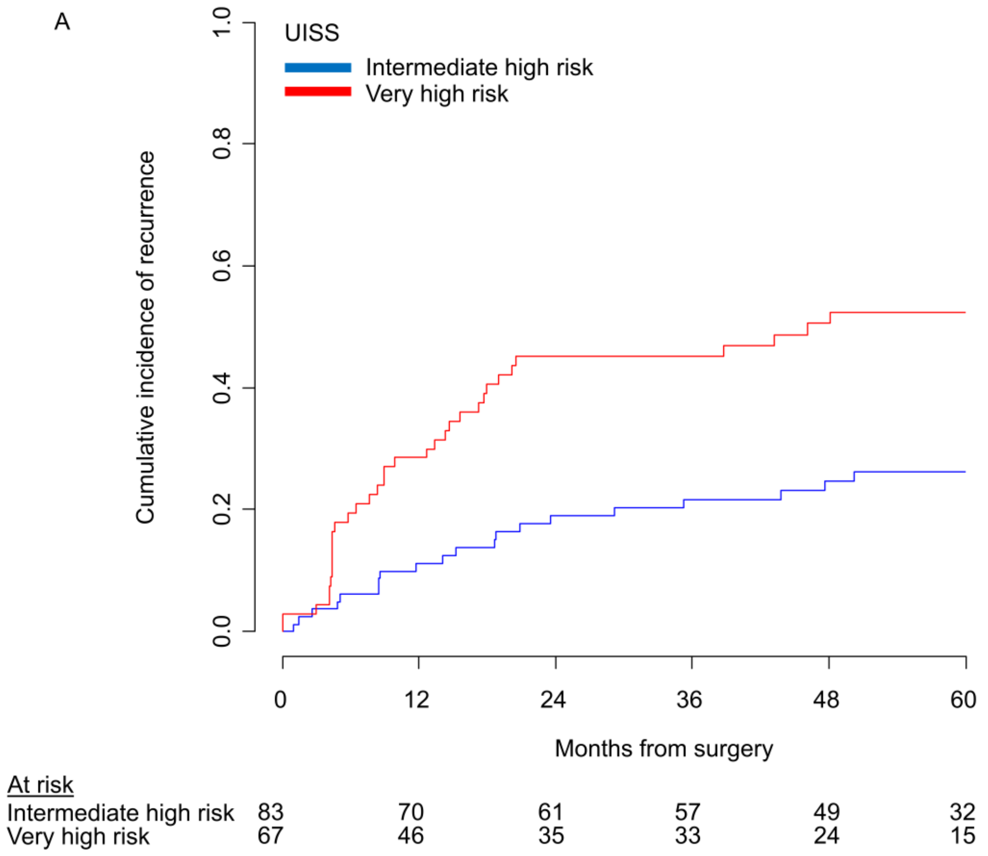

B

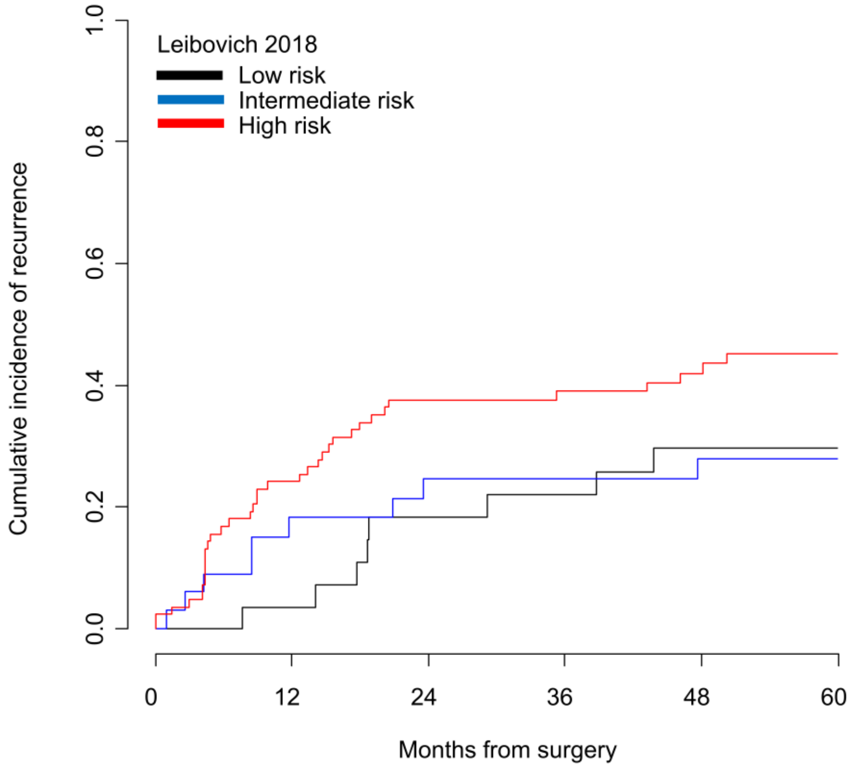

At risk						
Low risk (group 1)	29	27	22	21	16	11
Intermediate risk (group 2)	33	26	24	24	20	11
High risk (group 3)	84	61	48	43	36	24

C

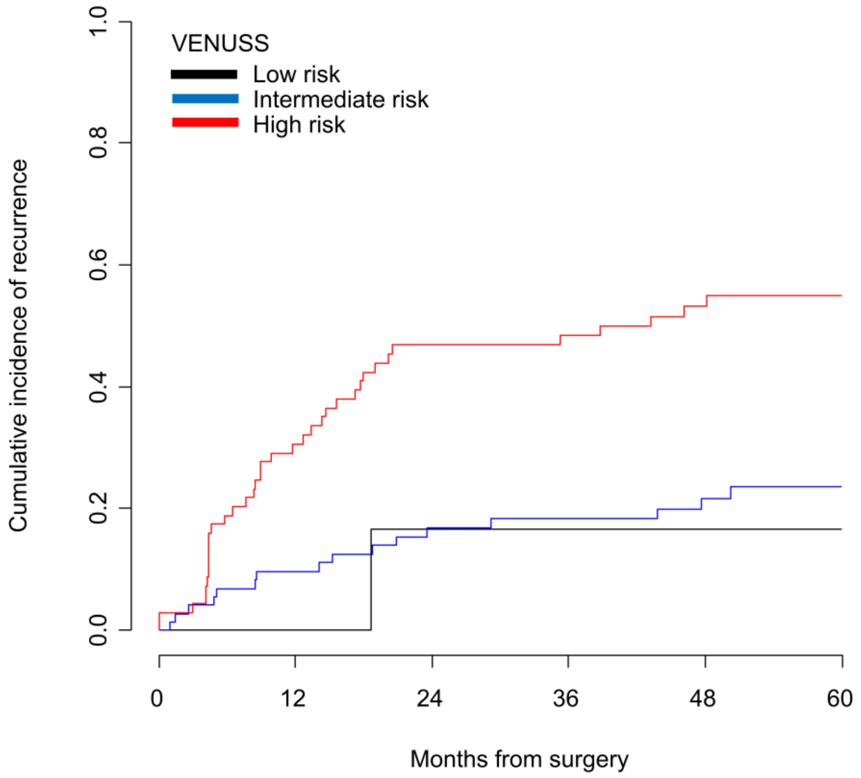

At risk						
Low risk	6	6	5	4	4	3
Intermediate risk	75	64	56	53	43	27
High risk	69	46	35	33	26	17
